# Arbutin Protects Retinal Pigment Epithelium Against Oxidative Stress by Modulating SIRT1/FOXO3a/PGC-1α/β Pathway

**DOI:** 10.3389/fgene.2022.922807

**Published:** 2022-08-16

**Authors:** Han Tang, Han Du, Xielan Kuang, Hao Huang, Jingshu Zeng, Chongde Long, Binbin Zhu, Licheng Fu, Hua Wang, Qingjiong Zhang, Shuibin Lin, Jianhua Yan, Huangxuan Shen

**Affiliations:** ^1^ State Key Laboratory of Ophthalmology, Zhongshan Ophthalmic Center, Sun Yat-sen University, Guangzhou, China; ^2^ Biobank of Eye, State Key Laboratory of Ophthalmology, Zhongshan Ophthalmic Center, Sun Yat-sen University, Guangzhou, China; ^3^ Department of Ophthalmology, The First Affiliated Hospital of Guangzhou Medical University, Guangzhou, China; ^4^ Department of Intensive Care, Zhujiang Hospital, Southern Medical University, Guangzhou, China; ^5^ Center for Translational Medicine, Precision Medicine Institute, The First Affiliated Hospital, Sun Yat-sen University, Guangzhou, China

**Keywords:** age-related macular degeneration, oxidative stress, SIRT1 signaling pathway, mitochondrial health, cell senescence

## Abstract

Age-related macular degeneration (AMD), which is the leading cause of blindness among the elderly in western societies, is majorly accompanied by retinal pigment epithelium (RPE) degeneration. Because of the irreversible RPE cell loss among oxidative stress, it is crucial to search for available drugs for atrophic (dry) AMD. RNA-Seq analysis revealed that genes related to aging and mitochondrial health were differentially expressed under Arbutin treatment, whereas compared to oxidative injury, our study demonstrated that Arbutin substantially abrogated oxidative stress-induced cell senescence and apoptosis linked to intracellular antioxidant enzyme system homeostasis maintenance, restored mitochondrial membrane potential (MMP), and reduced the SA-β-GAL accumulation in RPE. Furthermore, Arbutin alleviated oxidative stress-mediated cell apoptosis and senescence *via* activation of SIRT1, as evidenced by the increase of the downstream FoxO3a and PGC-1α/β that are related to mitochondrial biogenesis, and the suppression of NF-κB p65 inflammasome, whereas rehabilitation of oxidative stress by SIRT1 inhibitor attenuated the protective effect of Arbutin. In conclusion, we validated the results in an *in vivo* model constructed by NAIO_3_-injured mice. OCT and HE staining showed that Arbutin sustained retinal integrity in the case of oxidative damage *in vivo*, and the disorder of RPE cytochrome was alleviated through fundus observation. In summary, our findings identified that oxidative stress-induced mitochondrial malfunction and the subsequent senescence acceleration in RPE cells, whereas Arbutin inhibited TBHP-induced RPE degeneration *via* regulating the SIRT1/Foxo3a/PGC-1α/β signaling pathway. These findings suggested that Arbutin is a new agent with potential applications in the development of AMD diseases.

## Introduction

Age-related macular degeneration (AMD) is a leading cause of progressive loss of central vision resulting in permanent visual impairment and even legal blindness (Mitchell P et al., 2018). The dry form of AMD (known as atrophic AMD), which in advanced forms involves geographic atrophy of the retinal pigment epithelium (RPE), accounts for ∼90% of all AMD cases(Evans and Syed, 2013). As a highly specialized cell adjacent to photoreceptors on the apical side, with Bruch’s membrane and choriocapillaris on the basal side, RPE plays a vital role in retinal metabolism and the transportation of nutrients such as phagocytosis and clearance of photoreceptor outer segments, light absorption, and heat exchange (Datta et al., 2017). Attributable to aging and the accumulated impacts of environmental stresses, RPE under prolonged metabolic burden will induce oxidative stress, which is considered to be a major contributor to the RPE in AMD pathology.

RPE cells that are constantly exposed to oxidative stress during aging may cause hazardous metabolites accumulation in mitochondria (Kaarniranta et al., 2020), disruption of mitochondrial structure, and mtDNA mutations, leading progressively to cell senescence (Blasiak et al., 2013). In recent years, a study has confirmed that mitochondria in RPE are a site of primary pathology in dry AMD through analyzing human donor tissue (Fisher and Ferrington, 2018). In consideration of the critical role of mitochondria in RPE degeneration and AMD pathology, it is a promising therapeutic strategy to exploit drug that targets mitochondrial health in AMD treatment.

Plenty of natural ingredients were confirmed to show therapeutical effects on AMD, for example, quercetin (Zhu et al., 2019), β-carotene (Chew et al., 2013), and zeaxanthin (Age-Related Eye Disease Study 2 Research Group, 2013). Our previous studies also found numerous components of Chinese medicine such as curcumin (Zhou et al., 2017), lutein (Liu et al., 2017), and 2,3,5,6-tetramethylpyrazine (Huang et al., 2021) exerted protective efficacy on RPE cells. Moreover, in terms of seeking proteins involved in AMD pathological, we elucidated the molecular mechanisms of ID2 underlying oxidative damage response in RPE cells (Fan et al., 2018). Arbutin is a plant-derived glycosylated hydroquinone that is mainly extracted from the leaves of bearberry (*Vaccinium vitis-idaea L*). It has been reported that Arbutin is a tyrosinase inhibitor that possesses antineurodegenerative activity by suppressing the conversion of l-tyrosine into l-DOPA(Ding et al., 2020; Jin et al., 2020). Arbutin also has been proven to possess therapeutic effects on various diseases (Saeedi et al., 2021). Therefore, the appliances of Arbutin upon diseases remains enormous potentials.

Although intravitreal injection of antivascular endothelial growth factor is the most widely used therapy aimed at neovascularization, there are still no disease-altering therapies or specific drugs available for dry AMD (Saini et al., 2017)(“Nicotinamide Ameliorates Disease Phenotypes in a Human iPSC Model of Age-Related Macular Degeneration—PubMed,” n. d.). Components derived from plants tend to exhibit smaller side effects than synthetic compounds, which means that Arbutin was highly suitable for further therapeutic assessment to explore the protective role of Arbutin on RPE.

Thus, this study conducted in-depth research to investigate the characteristics of Arbutin in improving RPE conditions targeting mitochondrial health and senescence under TBHP (tert-butyl hydroperoxide)-induced oxidative stress and attempted to illustrate the intrinsic mechanism.

## Materials and Methods

### Animals

C57/BL6J mice were purchased from Guangdong Medical Laboratory Animal Center (Foshan, China) and were maintained in the Ophthalmic Animal Laboratory, Zhongshan Ophthalmic Center, Sun Yat-sen University, Guangzhou, China. All mice were housed in an SPF environment with controlled humidity and temperature, exposed to 12-h light and 12-h dark cycle, and provided with a diet and water. C57/BL6J mice were composed of three female adults (27–30 g, 3 months of age) per cage. To establish an oxidative stress-induced retinal degeneration model, mice were consecutively injected with 35 mg/kg of NaIO_3_ through a tail vein for 7 days. Arbutin was administrated to mice through intraperitoneal injection for 14 days before and during the NAIO_3_ injection. The control mice were injected with the same volume of PBS. Optical coherence tomography T (Heidelberg Engineering, Germany) was conducted on the mice that experienced different procedures on days 14 and 21.

### Cell Culture and Treatment

Adult retinal pigment epithelial cell line-19 (ATCC, Virginia, USA, ARPE-19) was obtained from the American type culture collection. The cells were cultured in high glucose Dulbecco’s modified Eagle’s medium (DMEM, HyClone, GE Healthcare Life Sciences, United States) with 10% FBS (Gibco, Thermo Fisher Scientific, United States) and 1% of penicillin/streptomycin (HyClone, GE Healthcare Life Science, United States) at 37℃ with 5% CO_2_.

Human primary-RPE cells were isolated from the eyeballs of donors who died accidentally without ophthalmic diseases, who were 20–40 years old, from the Eye Bank of Guangdong Province (Zhongshan Ophthalmic Center, Sun Yat-sen University), adhering to the tenets of the Declaration of Helsinki. The isolation and culture of primary-RPE cells were performed as described previously (Fernandez-Godino et al., 2016). The isolated human primary-RPE cells were cultured in a Petri dish lined with BD Matrigel substrate membrane matrix. The culture medium contained 20% fetal bovine serum (GIBCO, Life Technology, United States) and 1% penicillin (100 μG/mL) and 1% streptomycin (100 μ DMEM/f12 (hyclone, GE Healthcare Lifescience, United States). In the following experiments, ARPE-19 cells and human primary-RPE cells were pretreated with or without Arbutin (Aladdin, China) of different concentrations for 24 h; next, after washing at least three times to eliminate the residual Arbutin, the cells were exposed to TBHP (tert-butyl hydroperoxide) in serum-free DMEM for another 24 h.

### Apoptosis, Cell Viability, and Commercial Reagents

In the presence or absence of TBHP and Arbutin, ARPE-19 cells and human primary-RPE cells were collected for the assessment of apoptosis performed using Muse Annexin V & Dead Cell Kit (Merck Millipore, Germany), and the cell viability was determined using the Cell Counting Kit-8 (CCK8, Dojindo, Japan). Sirtinol (Cat# 410536–97–9, Selleckchem), a selective inhibitor of SIRT1 activity, was dissolved in 0.004% DMSO and used at a final concentration of 10 μM.

### RNA-Seq Analysis

Three sets of sequencing were performed in this study. The three groups respectively were the whole RNA sequencing of oxidative damaged ARPE-19 cells. Control cells and oxidative damaged ARPE-19 cells with Arbutin pretreatment.

Total RNA of ARPE-19 cells was extracted from the cells with Trizol reagent (Invitrogen, Thermo Fisher Scientific, New York, United States). Whole-transcriptome sequencing (RNA-seq) was carried out at CapitalBio, Beijing. After ribosomal RNA was depleted, sequencing libraries were constructed following the standard Illumina protocols and were subsequently processed by a Hiseq2000 sequencing system (125 bp paired-end reads, four samples per lane; Illumina). Data were analyzed using a previously published pipeline. After quality control, reads were aligned to the human transcriptome (GRCh38; Ensembl.org; in the public domain) and quantified using a STAR aligner. DESeq2 (Version 1.12.4) was used to determine differentially expressed genes between two samples. Genes were considered as expressed significantly different if q < 0.001 and foldchange > 1.5.

### Reverse Transcription–Quantitative Polymerase Chain Reaction and Western Blotting

Real-time PCR was conducted as previously described (He et al., 2017). The primers were synthesized by Sangon Biotech (Guangzhou, China). Primer sequences were as follows**:** SIRT1_F: TAT​ACC​CAG​AAC​ATA​GAC​ACG​C and SIRT1_R: CTCTGGTTTC ATGATAGCAAGC; NFKB1_F: GAG​ACT​TTC​GAG​GAA​ATA​CCC​C and NFKB1_R: GTA​GCC​ATG​GAT​AGA​GGC​TAA​G; FOXO3_F: AGC​CGA​GGA​AAT​GTT​CGT​C and FOXO3_R: CCT​TAT​CCT​TGA​AGT​AGG​GCA​C; PPARGC1B_F: GGAGGAGGAGGA GGACGATGAAG and PPARGC1B_R: TGC​TTG​GTG​GGC​TCT​GGT​AGG; PPARGC1A_F: CAG​AGA​GTA​TGA​GAA​GCG​AGA​G and PPARGC1A_R: AGC​ATC​ACA​GGT​ATA​ACG​GTA​G.

Total protein concentration was determined using a bicinchoninic acid (BCA) assay (cat# 23,227, BCA Protein Assay Reagent Kit, Thermo Fisher Scientific Pierce Protein Research Products, Rockford, Illinois, United States). The western blotting assay was conducted as previously described (Zhang H. et al., 2020). The primary antibodies were as follows: SIRT1 (CST, New York, United States, #9475, 1:1000), p44/42 MAPK (Erk1/2) (CST, New York, United States, #9102, 1:1000), FOXO3 (Boster Biological Technology, United States, Pleasanton, PB9196, 1:1000), PPARGC1A (Boster Biological Technology, United States, Pleasanton, BM4898, 1:1000), PPARGC1B (Boster Biological Technology, United States, Pleasanton, A02933-1, 1:1000), NF-κB1 p105/p50(CST, New York, United States, #13586 1:1000), and Phospho-NF-κB p65 (Ser536) (CST, New York, United States, #3033 1:1000).

### SA-β-GAL Staining Activity

SA-β-gal was detected using an *in situ* β-Galactosidase Staining Kit (Beyotime, RG0039, Beijing, China). ARPE-19 and human primary-RPE cells of each group were plated in each well (9.6 cm^2^) of six-well plates overnight. After experimental treatment, cells were fixed with a fixative solution for 10–15 min and washed twice with PBS, and then incubated in SA-β-Gal working solution at 37℃ for 24 h. The SA-β-GAL^+^ cells with blue perinuclear staining were observed using a microscope.

### Determination of the Glutathione/Superoxide Dismutase/Malondialdehyde Activity

The cells were seeded into six-well plates at a density of 15 × 10^4^ cells/well and incubation was carried out as described in cell cultures. Following tryptic digestion, cells suspended in PBS were then frozen in liquid nitrogen and thawed at 37℃. Lysates were centrifuged at 1000 rpm for 10 min and the supernatant of each group was collected. The concentrations of glutathione (GSH), superoxide dismutase (SOD), and malondialdehyde (MDA) were quantified using GSH and GSSG Assay Kit following the manufacturer’s instruction (Beyotime Institute of Biotechnology, Nanjing, China), SOD Assay Kit (Beyotime Institute of Biotechnology, Nanjing, China), and Lipid Peroxidation MDA Assay Kit (Beyotime Institute of Biotechnology, Nanjing, China).

### Wound Healing Assay

ARPE-19 and primary-RPE cells were cultured in 12-well plates cultured to 80% confluence and wounds were made with a standard 200-µL pipette tip. First, we used a ruler to compare and a marker pen to mark the culture plate by drawing a horizontal line evenly on the back of the six-well plate, approximately every 0.5∼1 cm, across the holes, and then added approximately 5 × 10^5^ cells. The principle of inoculation was that the fusion rate reached 100% overnight. The next day, we used a gun head to scratch the cell layer perpendicular to the cell plane and perpendicular to the line drawn on the back of the plate the previous day. Photos were taken at specific positions after scratching (0 h) and 24 and 48 h after scratching. The wound area was measured using the program Fiji (ImageJ-win 64; https://imagej.net/Fiji) and was set in relation to the wound area at the respective point at 0 h.

### Mitochondrial Membrane Potential (ΔΨm)

ΔΨm staining was performed using Mitochondrial Membrane Potential Kit (Sigma Aldrich, United States), 5000 ARPE-19 cells/well were seeded in 96-well plates (Corning, United States) and treated with Arbutin and TBHP for certain hours, and 100 μL of JC-1 solution was added to the cells for 20 min. The absorbance at 514/529 and 585/590 nm were detected using a microplate reader (BioTek Instruments, Winooski, Vermont, United States), and cells were observed under a fluorescent inverted microscope.

### Immunofluorescence

Immunofluorescence staining was used to detect RPE65 expression. For cell slides, cells were fixed with 4% paraformaldehyde for 15 min, washed three times in 1 × PBS, and then repaired with 1 × Cell Antigen Repair Solution (Beyotime, China) for 5 min at room temperature. Cells were observed under a fluorescent inverted microscope.

### Statistical Analysis

All data were expressed as mean ± SD of at least three independent experiments. Statistical analysis was performed using GraphPad Prism 8.0 statistical software. Statistical differences were determined with a one-way analysis of variance and LSD test; *p* values of <0.05 were considered statistically significant.

## Results

1 Arbutin Protected ARPE-19 and Human Primary Retinal Pigment Epithelium Cells Against TBHP-Induced Apoptosis and Senescence

**FIGURE 1:**
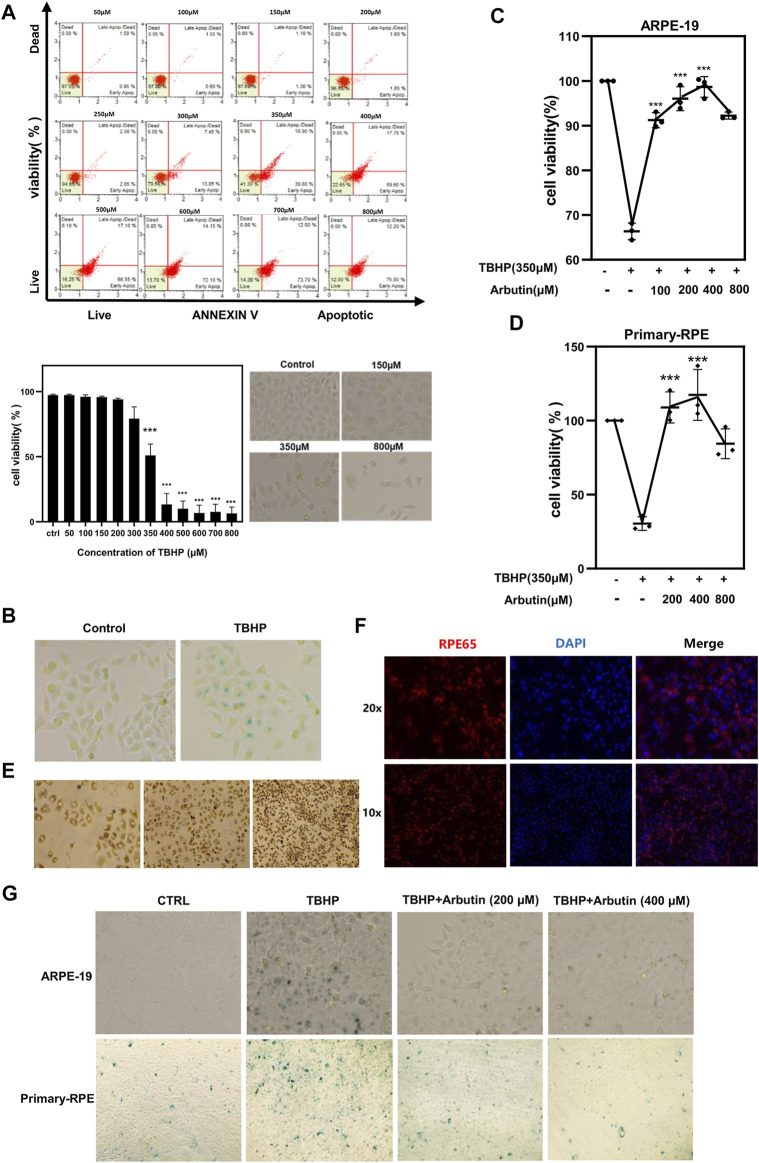
Arbutin protected retinal pigment epithelium (RPE) cells from tert-butyl hydroperoxide (TBHP)-induced senescence and apoptosis. **(A)** RPE cells were treated with different concentrations of TBHP ranging from 50 µM to 800 µM for 24 h and cell viability was determined with Annexin V & Dead. TBHP inhibited RPE cell viability and the apoptosis ratio showed a statistical difference at a concentration of 350 µM. RPE cell morphology displayed abnormality under a microscope at concentrations of 150, 350, and 800 µM. **(B)** 350 µM TBHP treatment for 24 h upregulated SA-β-Gal activity in RPE cells. **(C)** flow cytometric analysis and **(D)** CCK8 assay respectively showed that Arbutin pretreatment rescued ARPE-19 and primary-RPE cells from TBHP-induced apoptosis in a concentration-dependent manner while being unable to obtain stronger protective effectiveness at the highest concentration of 800 µM. **(E)** cell confluence and morphology of human primary-RPE cells isolated from the eyeballs of donors. **(F)** immunofluorescence shows the expression of RPE65 (RPE-specific protein) in primary-RPE cells. **(G)** ARPE-19 and primary-RPE cells subjected to TBHP showed a significant increase in SA-β-gal expression, which is decreased in cells incubated with Arbutin prior to TBHP damage. (*p < 0.05, **p < 0.01, ***p < 0.001, *n* = 3, bars represent SD).

To induce oxidative stress and cell senescence, ARPE-19 cells were subjected to increased concentrations of TBHP (50–800 µM) for 24 h. Cell viability assessed using Annexin V & Dead Cell Kit confirmed that exposure to 350 µM TBHP led to apparent cellular changes and approximated 50% apoptosis of ARPE-19 (Figure 1A). Furthermore, β-galactosidase staining showed that TBHP treatment contributes to SA-β-gal accumulation in ARPE-19 cells (Figure 1B). Human primary-RPE cells isolated from the eyeballs of donors possess normal cell confluence and morphology and were confirmed *via* RPE65 protein staining (Figures 1E,F). As 350 µM of TBHP had the most significant effect on cells, that concentration was used for all further experiments. Next, we pretreated ARPE-19 and human primary-RPE cells with gradient concentrations of Arbutin (100–800 µM) for 24 h and found that the TBHP-induced apoptosis could be rescued using Arbutin in a dose-dependent manner (100–400 µM) (Figures 1C,D) and the SA-β-gal level showed prominently decrease compared to damaged cells (Figure 1G). Taken together, the results demonstrated that Arbutin displays effective protection of RPE cells among TBHP-induced apoptosis and senescence.

2 Arbutin Plays a Protective Role by Affecting Genes Associated With Cell Aging and Mitochondrial

**FIGURE 2:**
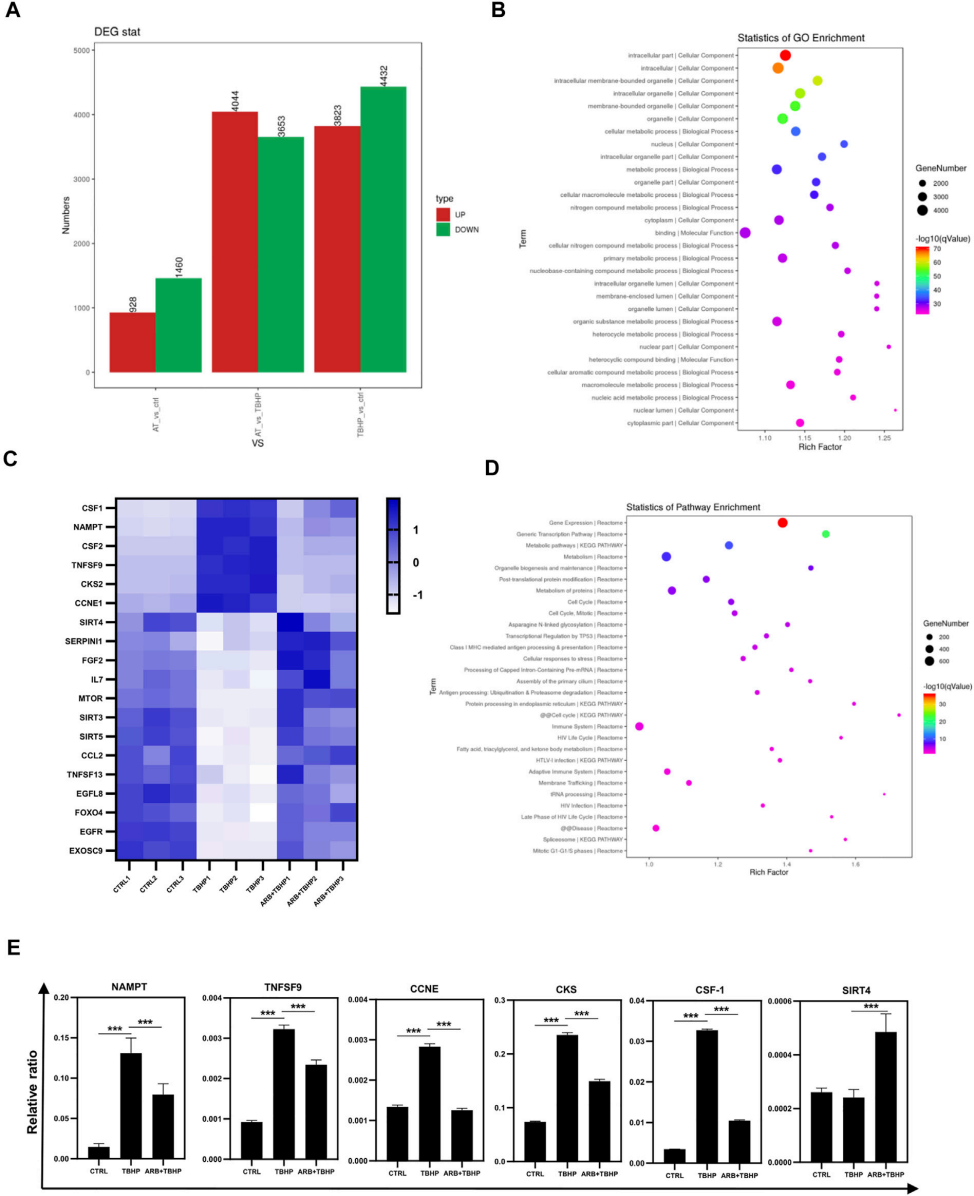
Transcriptome sequencing (RNA-seq) of ARPE-19 cells protective by Arbutin. **(A)** the number of differentially expressed genes among the control, TBHP injury, and Arbutin pretreatment groups (AT refers to Arbutin and TBHP). **(B)** GO pathway enrichment results of differential genes. **(C)** heatmap of differential expression of genes related to cell aging and mitochondrial health in different experimental groups. **(D)** pathway enrichment results of partially differential genes coincided with heatmap. **(E)** RT-PCR results of genes related to cell aging and mitochondrial health in different experimental groups (*p < 0.05, **p < 0.01, ***p < 0.001, *n* = 3, bars represent SD).

To elucidate the internal mechanism of Arbutin protecting ARPE-19 cells from oxidative stress, we collected the total mRNA of ARPE-19 cells treated by different experimental groups and applied RNA sequencing. RNA-seq identified a large number of differentially regulated genes (Figure 2A) related to cell senescence and mitochondrial health (Figure 2C), which were preliminarily verified *via* RT-PCR and coincided with the results of heatmap (Figure 2E). Gene ontology analyses of biological processes highlighted the expected changes following the maintenance of cell metabolism and mitochondrial function (Figures 2B,D).

3. Arbutin Involved in Maintaining Intracellular Antioxidant Enzyme Homeostasis and Restoring Mitochondrial Membrane Potential

**FIGURE 3:**
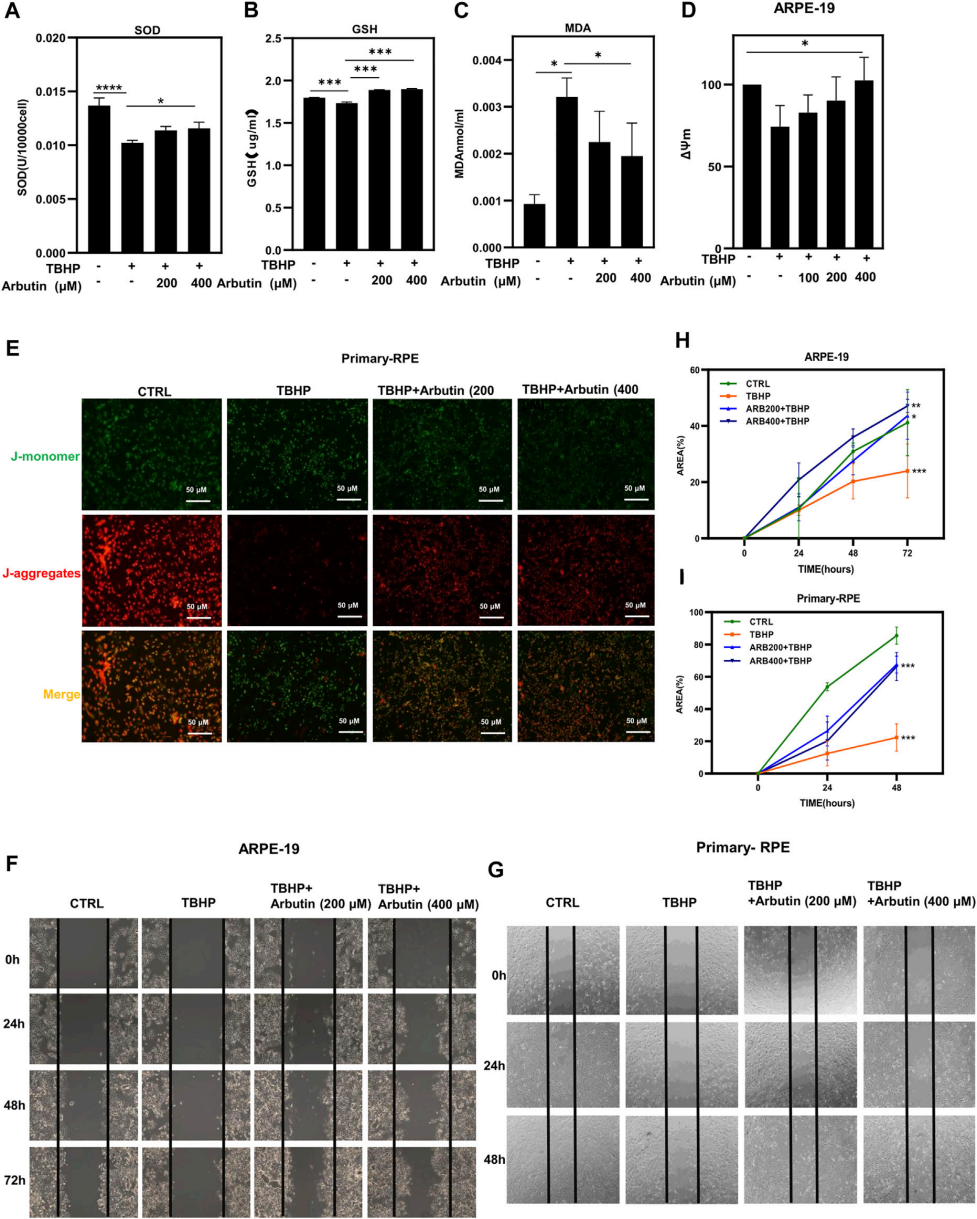
Arbutin improved cellular functions of RPE cells under oxidative stress. **(A)** antioxidant enzyme level analysis of SOD in ARPE-19 cells during oxidative damage while with or without Arbutin pretreatment. **(B)** glutathione peroxidase activity was detected. **(C)** malondialdehyde (MDA) was measured in different ARPE-19 cell treatments. **(D)** the statistical histogram of mitochondrial functional examination showed that 100 mm, 200 μM and 400 μM Arbutin promoted the ΔΨm of ARPE-19 cells during TBHP treatment. **(E)** fluorescence images collected after JC1 staining show that Arbutin restores JC-1 aggregates/monomer ratio in primary-RPE cells. **(F)** ARPE-19 cells were seeded in a 24-well plate and applied wounds at the confluence of 80%. The cells were pretreated with or without Arbutin and then subjected to TBHP (350 µM). Photos were taken at different time points post distinct treatments. **(G)** human primary-RPE cells planted in a 24-well plate and created wounds at the density of 80%. The cells were pretreated with or without Arbutin and then subjected to TBHP (350 µM). **(H)** the area of ARPE-19 cells situated amid the two black lines was calculated from images. **(I)** the area of human primary-RPE cells situated amid the two black lines was calculated from images. (*p < 0.05, **p < 0.01, ***p < 0.001, *n* = 3, bars represent SD).

To further evaluate the protective effects of Arbutin on RPE cells under oxidative stress conditions, we first detected GSH and SOD, two key members of the antioxidant system in ARPE-19 cells, which are responsible for detoxification and tend to cause cell resistance to peroxisome stimulated by TBHP. The assay revealed that Arbutin pretreatment efficiently re-upregulated intracellular GSH and SOD activity in a dose-dependent manner in the presence of TBHP (Figures 3A,B). Next, we examined MDA, an end product of MDA lipid oxidation, which affects mitochondrial respiratory chain complexes and key enzyme activities in mitochondria *in vitro*. Arbutin treatment (at a concentration of 200 µM and 400 µM) significantly reduced the MDA activities of ARPE-19 cells at 24 h (Figure 3C), compared to that of the TBHP group. Because of elevated oxygen pressure, the mitochondria in injured RPE cells are prone to compromised energy production, resulting in MMP reduction, leading to RPE dysfunction and death.

To clarify whether Arbutin could alleviate RPE mitochondrial damage, we performed ΔΨm staining. ARPE-19 cells were pretreated with Arbutin at gradient concentrations (100/200/400 µM) for 24 h; then, we used 350 µM TBHP to cause a reduction in MMP, and the J-aggregates/J-monomer ratio was dramatically decreased in the TBHP-only group but was largely preserved in the Arbutin pretreatment group (Figure 3D). Human primary-RPE cells were subjected to the same treatment as ARPE-19 cells. Similar results were obtained when observed using a fluorescent inverted microscope (Figure 3E). These results illustrated that Arbutin preserved RPE cellular functions under oxidative stress by maintaining mitochondrial health. Migration is an essential function of RPE cells, we performed wound healing to assess the effects of Arbutin on RPE migration under TBHP damage. ARPE-19 and human primary-RPE cells were planted in 12-well plates and pretreated with Arbutin at two concentrations (200/400 µM) for 24 h; then, we applied 350 µM TBHP to form RPE cell wound healing impairment. Wounds were scratched before TBHP treatment, and the FBS was reduced to 3% throughout the experiment. Photos were taken at 0, 24, 48, and 72 h (Figure 3F), and the area occupied by the cells was calculated within each photo (Figure 3G). We found that migration function disturbance of ARPE-19 cells induced by TBHP could be rehabilitated using Arbutin in a dose-dependent manner. Similar results were obtained in human primary-RPE cells (Figures 3H,I). These results indicated that Arbutin exerted efforts in maintaining normal RPE cell migration capability.

4 Arbutin Protected Retinal Pigment Epithelium Against Oxidative Stress Through the SIRT1/FOXO3A/PGC-1α/β and NF-κb/P65 Signaling Pathway

**FIGURE 4:**
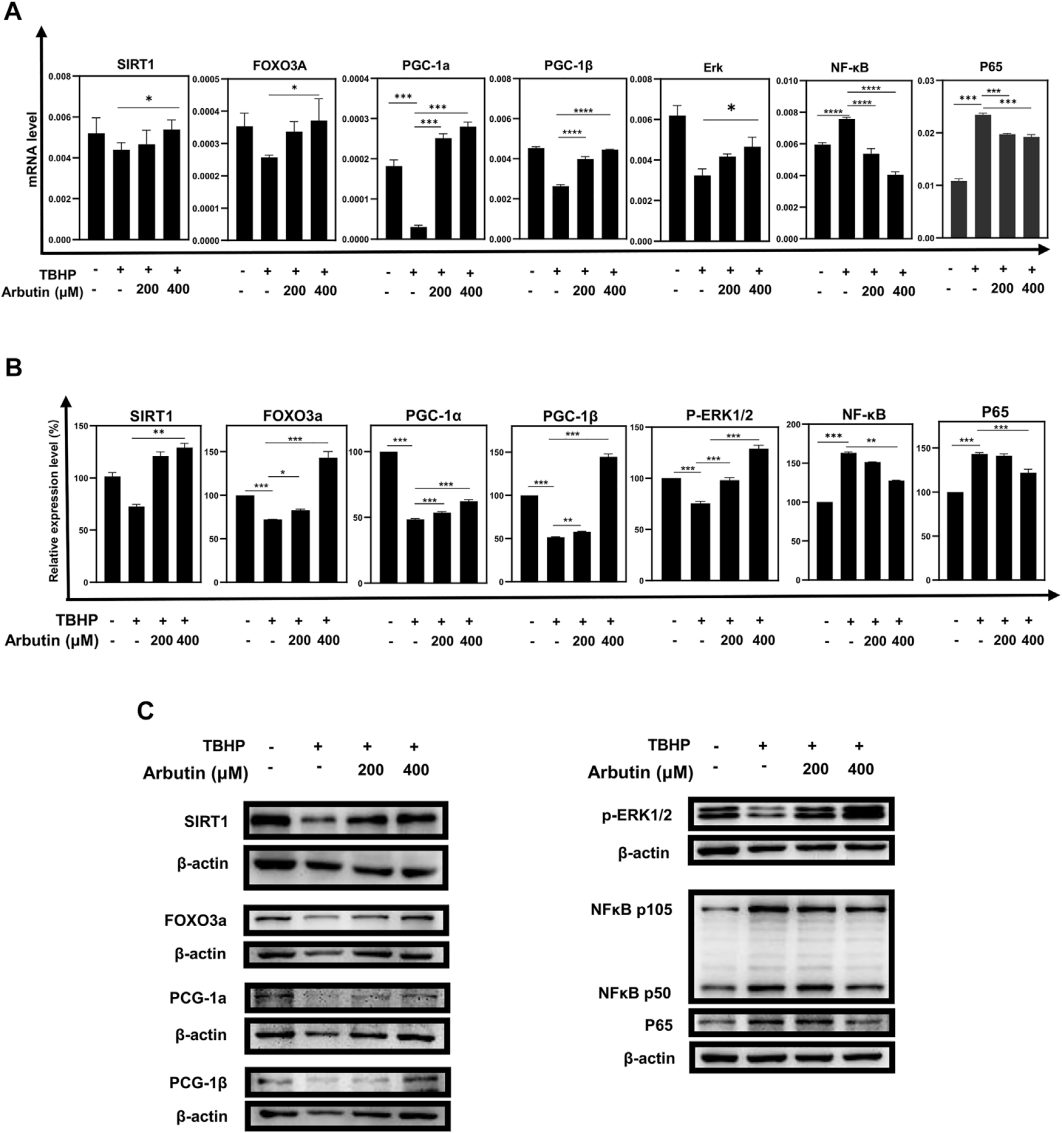
Arbutin exerted protective effects *via* the SIRT1/FOXO3A/PGC-1α/β and NF-κB/p65 signaling pathway. **(A)** qRT-PCR was used to measure transcript levels of the SIRT1/FOXO3a/PGC-1α/β pathway and NF-κB/p65 genes. TBHP decreased the expression of SIRT1, FOXO3a, and PGC-1α/β and increased the expression of NF-κB/p65, whereas mRNA levels in the groups that were pretreated with Arbutin showed reversed trend. **(B)** western blots were conducted to detect the proteins level of SIRT1, FOXO3a, PGC-1α/β, p-ERK, and NFKB1/P65. **(C)** imageJ was used to analyze the relative expression level of the proteins mentioned above (*p < 0.05, **p < 0.01, ***p < 0.001, *n* = 3, bars represent SD).

Since SIRT1 has been reported to involve in the regulation of cellular senescence and mitochondrial dysfunction in retinal degeneration diseases, to elucidate the protection mechanism of Arbutin in ARPE-19 cells under oxidative stress injury, we examined whether Arbutin upregulated SIRT1 and Forkhead box O3a (FOXO3a), peroxisome proliferator-activated receptor–gamma coactivator-1α/β (PGC-1α/β) that played essential role amid mitochondrial biogenesis. We found that while the mRNA levels of SIRT1, FOXO3a, PGC-1α, and PGC-1β decreased, the mRNA levels of NFKB1 and P65 increased in the TBHP-only group (Figure 4A). In the Arbutin pretreatment group, the alteration of the above-mentioned genes’ mRNA expression level caused by TBHP could be reversed (Figure 4A). At the protein level, western blot analysis of SIRT1, FOXO3a, PGC-1α, PGC-1β, NFKB1, and P65 show the corresponding results with RT-PCR (Figures 4B,C). These results indicated that the activity of the SIRT1/FOXO3A/PGC-1α/β pathway was inhibited in oxidative stress, whereas in Arbutin pretreated RPE cells, this pathway could be upregulated. Moreover, TBHP activated the pro-inflammatory protein nuclear factor κB (NF-κB) and P65 related to a plethora of inflammatory reactions, which were also reversed by Arbutin.

5 Inhibition of SIRT1 Undermined the Protective Effects of Arbutin on Stressed ARPE-19 Cells.

**FIGURE 5:**
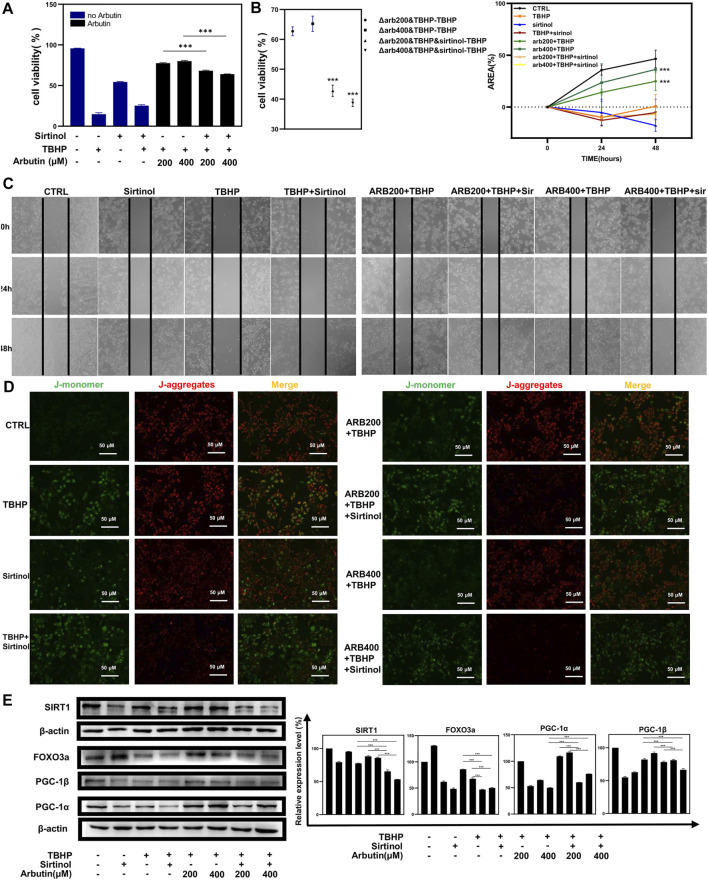
Sirtinol diminished the capability of Arbutin to assist ARPE-19 cells to defend against oxidative stress. **(A) (B)** flow cytometric analysis showed that cells treated with only sirtinol, TBHP, cotreated with sirtinol, and TBHP displayed decreased cellular viability. However, sirtinol conduction diminished the protective capacity of Arbutin. **(C)** ARPE-19 cells were seeded in a 24-well plate and applied wounds at the confluence of 80%. The cells were pretreated with or without Arbutin and then subjected to TBHP (350 µM); meanwhile, cells in certain groups were incubated with sirtinol. Photos were taken at different time points post distinct treatments. **(D)** fluorescence images observed that ARPE-19 treated with Arbutin while subjected to sirtinol and then exposed to TBHP was unable to recuperate ΔΨm. **(E)** with sirtinol administration, the protein levels of the SIRT1/FOXO3a/PGC-1α/β pathway decreased in the presence of Arbutin (*p < 0.05, **p < 0.01, ***p < 0.001, *n* = 3, bars represent SD).

To corroborate that Arbutin exerted a protective effect by activating SIRT1 in RPE cells, we used sirtinol (10 μM) to provide sufficiently inhibitory effects on SIRT1 (Hubbard et al., 2013). Next, APRE19 cells experienced treatment of Arbutin (200/400 μM) for 24 h and were then incubated with TBHP for 24 h. Apoptotic assays revealed significant differences between groups that administered sirtinol or not, illustrating that the inhibition of SIRT1 diminished the capability of Arbutin assisting ARPE-19 cells to defend against oxidative stress to some extent. (Figures 5A,B). In addition, wound healing assays showed that after sirtinol administration, Arbutin was unable to recover the migration ability of ARPE-19 under TBHP exposure (Figure 5C). Similar results were obtained when detecting MMP (Figure 5D). Furthermore, we harnessed western blot analysis to clarify that Arbutin acted on SIRT1 to modulate the SIRT1/FOXO3a/PGC-1α/β pathway; the results revealed that ARPE-19 cells incubated with sirtinol, TBHP, or both of them displayed evident suppression of SIRT1 and the downstream proteins when compared with the control group or cells pretreated with Arbutin. Meanwhile upregulating of SIRT1/FOXO3a/PGC-1α/β axis in cells pretreated with Arbutin could not be observed with the administration of sirtinol (Figure 5E). These results demonstrated that SIRT1 is an indispensable molecule when Arbutin manifested cellular protective effects.

6 Arbutin Mitigated NaIO_3_ Intravenous Injection-Induced Retinal Degeneration in C57BL/6J Mice

**FIGURE 6:**
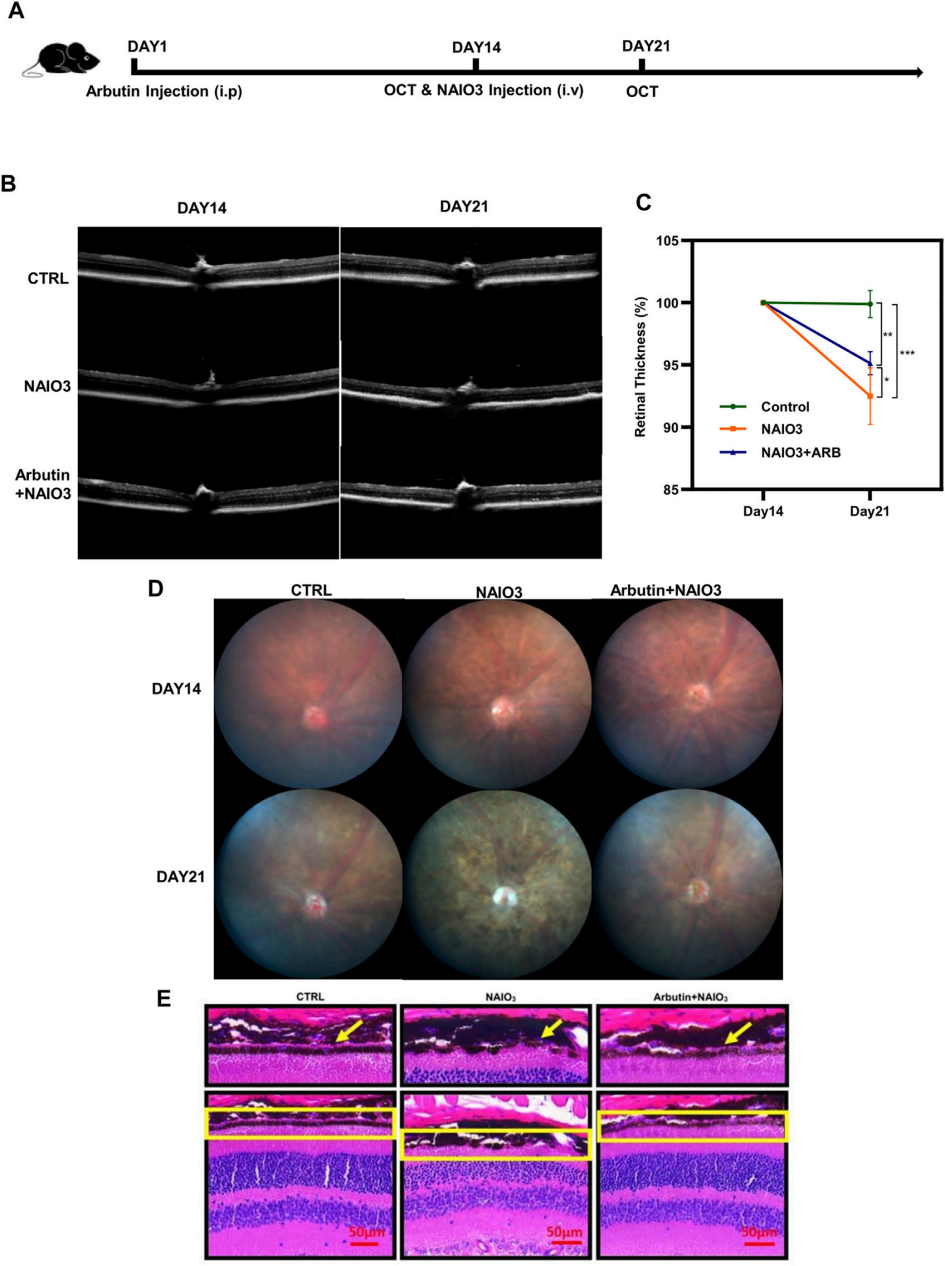
Arbutin attenuated NAIO_3_-induced retinal thickness loss in C57BL/6J mice. **(A)** experimental procedure: The experiments were based on wild-type C57/BL6 mice. **(B)** the images of retinas were taken by optical coherence tomography examination at the timepoints of days 14 and 21. **(C)** the retinal thickness difference of mice with different treatments is shown in the charts. **(D)** fundus imaging of mice in different groups. **(E)** H & E staining showed retinal sections of mice treated with different treatments (*p < 0.05, **p < 0.01, ***p < 0.001, *n* = 3, bars represent SD).

To further validate whether Arbutin provided protection *in vivo*, we established a mice model *via* intravenous injection of NaIO_3_. In subsequent experiments (Figure 6A), the retinas of mice were primarily recorded using OCT images after anesthesia. Results showed that compared with the normal mice (the control group), the retinas of the mice with NaIO_3_ injection (the model group) were obviously thinner. The retinas of the Arbutin preadministration group, sequentially injected with Arbutin and NAIO_3_, were slightly thinner than those of normal mice but thicker than those injured by NaIO_3_ (Figure 6B). Quantification of retinal thickness confirmed that Arbutin mitigated retinal damage caused by NaIO_3_ (Figure 6C). Compared with normal mice, Arbutin also alleviated the obvious thinning of the retina and the disorder of RPE cytochrome after NaIO_3_ treatment (Figure 6D). H & E staining of mouse eyeball paraffin sections showed that Arbutin reduced the damage to the RPE cell layer of mouse retina caused by NaIO_3_ (Figure 6E).

## Discussion

It is believed that oxidative damage is a major contributor to the pathogenesis of aging. TBHP exposure has been demonstrated to initiate oxidative stress reactions, leading to cellular antioxidant enzymes activities depletion (Roy and Sil, 2012). TBHP incubation disrupted the junctional integrity of the RPE and the oxidation of glutathione and thereby inducing membrane leakage leading to apoptosis (Rabin et al., 2012). Moreover, sodium iodate (NaIO_3_) selectively induced RPE cell death *via* oxidative damage making it widely used to construct reproducible animal models of RPE dystrophy and AMD. Based on that, we built a TBHP-induced cellular injury model and NAIO_3_-induced *in vivo* model to mimic the condition of RPE under oxidative stress in the progression of AMD.

TBHP increased cells stained positive for senescence-associated beta-galactosidase (SA-β-Gal) in a rat model (Yeh et al., 2020), and it had been proved that high glucose-induced RPE cell senescence could be validated by SA-β-Gal staining (Chen et al., 2019) (“Oxidative stress mediated by lipid metabolism contributes to high glucose-induced senescence in retinal pigment epithelium—PubMed,” n. d.). Therefore, we detected the activity of SA-β-gal to characterize cellular senescence (Gao et al., 2021). The results revealed that Arbutin alleviated the proaging effects of TBHP-triggered SA-β-gal production. The mitochondrial theory is one of the most prevalent theories of aging-related diseases, which claimed that under oxidative stress, mitochondrial nucleic acids, proteins, and membrane lipids are all subjected to redundant ROS attack, leading to dysfunctional or defective mitochondria, which ultimately contributes to age-related physiological function decline (Liang and Godley, 2003) (“Oxidative stress-induced mitochondrial DNA damage in human retinal pigment epithelial cells: a possible mechanism for RPE aging and age-related macular degeneration—PubMed,” n. d.). Mitochondrial disfunction causes the depletion of MMP (△ψm) (Chong and Zheng, 2016). In this study, Arbutin rehabilitated J-aggregates/J-monomer ratio of RPE to a normal level compared to the TBHP-only group. The *in vitro* results provided new insights into the potential of Arbutin in the regulation of oxidative stress triggered RPE degeneration by virtue of the antiaging effect and mitochondrial function amelioration. The protective effects were also confirmed in human primary-RPE cells and C57BL/6J mice.

SIRT1 (Sir2) is a class III histone deacetylase that is deeply involved in an extensive range of biological events, containing immune response, metabolism, and aging. Numerous studies demonstrated that SIRT1 showed deficiency in various organs such as the liver, heart, lung, and kidney in the course of senescence (D’Onofrio et al., 2018; Duan et al., 2021; Ryu et al., 2019). SIRT1 also functions as a cytoplasmic energy sensor of NAD+/NADH ratio to maintain mitochondrial metabolism homeostasis. Chaoting (Ma et al., 2021) found that Arbutin inhibits RPE inflammation and apoptosis by enhancing autophagy *via* SIRT1. Our study revealed that TBHP-triggered oxidative damage promoted RPE cellular senescence and mitochondrial dysfunction, which were analogous to AMD progression, whereas Arbutin attenuated the hallmark of cellular senescence, SA-β-gal overexpression, and restored △ψm through upregulating SIRT1 expression. Then, we used sirtinol, the SIRT1 inhibitor, to confirm that the depletion of SIRT1 activity reversed the antiaging and mitochondrial protective effect of Arbutin. Previous studies indicated that the FOXO3A, PGC-1, and NF-κB were identified to be the downstream targets of SIRT1 (Steinmetz et al., 2017; Sun et al., 2018; Yu et al., 2018). Genomic analysis discovered that FOXO3A is a longevity-associated transcription factor in human aging (Joshi et al., 2017; Zhang W. et al., 2020). Meanwhile, PGC-1, as a key regulator of mitochondrial biogenesis and respiration, is proved to be a critical determinant of longevity in mammals (Rera et al., 2011). In our study, we found that TBHP downregulated SIRT1/FOXO3a/PGC-1α/β axis, which was reactivated by Arbutin, and sirtinol suppressed the modulation of Arbutin. Moreover, the inflammatory protein NF-κB activated using TBHP was also downregulated by Arbutin. Nevertheless, based on our results, we observed that inhibiting the SIRT1 pathway was unable to completely eliminate the effects of Arbutin, which means that the protective effect of Arbutin is not only through the SIRT1 signaling pathway. Hence, further ascertaining the other pathway that Arbutin acts on is essential for future studies and drug therapy development in the treatment of AMD.

In conclusion, the results revealed that Arbutin rescued RPE cells from oxidative stress *in vitro* and *in vivo*, subsequent mitochondrial dysfunction, and cellular senescence *via* the activated SIRT1/FOXO3a/PGC-1α/β pathway. Based on previous findings that Arbutin showed protective effects in terms of autophagy, our research has shed light on the novel mechanism of Arbutin and provided perspective on the application of Arbutin on retinal degeneration decease. All these results contributed to the discovery of new drugs to intervene in AMD.

## Data Availability

All data included in this study are available upon reasonable request by contact with the corresponding author.
